# Cellular PrPCWD deposition identified at the maternal–foetal interface of chronic wasting disease-infected cervids

**DOI:** 10.1099/jgv.0.002337

**Published:** 2026-09-18

**Authors:** Jesse Cole, Erin E. McNulty, Amy V. Nalls, Nathaniel D. Denkers, Gary L. Mason, Candace K. Mathiason

**Affiliations:** 1 Department of Microbiology, Immunology, and Pathology, Colorado State University, Fort Collins, Colorado, USA

**Keywords:** amplification-IHC (AMP-IHC), CWD, prions, uterine gland, vertical transmission

## Abstract

Vertical transmission has been identified as a viable route of transmission in the spread of chronic wasting disease (CWD), yet demonstration of trafficking mechanism(s) at the maternal–foetal interface has been limited. Conventional assays, including immunohistochemistry (IHC), Western blot and ELISA, often fail to detect low concentrations of the aberrant prion protein (PrP^CWD^). Contemporary *in vitro* amyloid conversion assays can detect low concentrations of prion seeding activity but lack the ability to localize prion seeds within specific cellular structures of tissues. In this study, we developed an enhanced immunohistochemical approach, as IHC uniquely enables cellular localization, to reveal prion deposition in maternal–foetal tissues from experimentally infected Reeves’ muntjac. After confirming improved sensitivity of amplification-IHC (AMP-IHC) in lymphoid tissues, we applied the assay to placentomes and uterine samples, comparing the results to matched tissue sections assessed by conventional IHC. AMP-IHC consistently detected PrP^CWD^ deposition that was undetected by conventional IHC, including its presence within the uterine glandular epithelium and glandular lumen. These observations provide the first histological evidence depicting prion association with uterine glands – structures responsible for secreting vital nutrients during early pregnancy. The presence of PrP^CWD^ in these structures supports the hypothesis that the developing conceptus can be exposed to prions *in utero*. Our findings expand upon earlier studies demonstrating vertical CWD transmission and highlight AMP-IHC as a valuable tool for identifying low-level prion accumulation in tissues where conventional IHC proves insufficient.

## Introduction

Chronic wasting disease (CWD) is an invariably fatal neurodegenerative prion disease affecting cervids (deer, elk and moose) that was first identified in captive deer in Colorado in the 1960s [1]. CWD has since been reported in 37 US states, 5 Canadian provinces, Scandinavia and Asia [2, 3]. CWD poses serious ecological and economic challenges in wild populations as prevalence can exceed 80%, threatening herd health and ecosystem stability [4]. In captive cervid settings, outbreaks of CWD result in substantial economic losses due to mandatory depopulation, herd movement restrictions and limitations on intrastate and interstate commerce. At a broader scale, cervid-related wildlife activities, including hunting and wildlife-associated recreation, contribute ~$447 billion annually to the US economy [5–7]. The continued geographic expansion of CWD across North America has the potential to disrupt these activities through increased regulatory oversight and reduced participation, underscoring that CWD represents not only a progressive and fatal neurodegenerative disease of cervids but also a condition with measurable economic relevance [5–7].

CWD is unique among prion diseases because of its highly efficient transmission, largely attributable to the shedding of infectious prions in multiple biological secretions (saliva, urine, faeces and blood) from infected cervids, which can be transmitted through direct animal-to-animal contact as well as indirectly via contamination of the environment [8]. While horizontal transmission is widely recognized as the primary mechanism driving CWD spread, a growing body of evidence indicates that vertical transmission also contributes to disease maintenance.

Experimental studies have demonstrated PrP^CWD^ deposition in tonsillar lymphoid tissue as early as 40 days post-parturition in offspring born to experimentally inoculated Reeves’s muntjac (*Muntiacus reevesi*), hereafter referred to as ‘muntjac’ [9]. This work identified amplifiable prions in multiple tissues associated with the maternal–foetal interface across different stages of gestation, suggesting that *in utero* transmission may occur throughout the course of maternal infection [9]. Similar findings were reported in free-ranging elk (*Cervus elaphus nelsoni*), where serial protein misfolding cyclic amplification (sPMCA) detected prion amplification in maternal–foetal interface tissues and indicated that PrP^CWD^ may be more widely distributed in peripheral tissues than previously recognized [10]. The infectious nature of prions within these tissues was later confirmed through bioassay and real-time quaking-induced conversion (RT-QuIC) using a cervidized mouse model [11]. Evidence of vertical transmission has now been reported in both captive and free-range naturally exposed white-tailed deer (WTD) (*Odocoileus virginianus*) using bioassay and *in vitro* amplification approaches [RT-QuIC, protein misfolding cyclic amplification (PMCA)] [12–14].

Vertical transmission of prions is not unique to cervids. Maternal-to-offspring transmission has been described in sheep affected by scrapie, transgenic bovidized mice expressing bovine spongiform encephalopathy and cheetahs with feline spongiform encephalopathy [15–18]. In humans, prion deposition has been detected in reproductive tissues and cord blood from cases of variant Creutzfeldt–Jakob disease [19, 20]. Despite the evidence of maternal prion transmission in several species, including both experimental and free-ranging cervid populations, the extent of transmission, cellular localization and underlying mechanisms of trafficking at the maternal–foetal interface remain poorly defined.

Conventional prion detection methods [immunohistochemistry (IHC), Western blot and ELISA] have been instrumental in advancing our understanding of CWD, including intra-host prion distribution and transmission dynamics. All three methods are effective in the diagnosis of well-established infections, yet can often fail to identify low-level prion accumulation in peripheral or reproductive tissues [4]. Contemporary prion *in vitro* amyloid/prion amplification assays, including sPMCA [21] and RT-QuIC [22], offer improved sensitivity, permitting detection of minute quantities of amyloid. However, neither method identifies the cellular localization of prion aggregation. Previous studies have demonstrated prion deposition within placentomes of sheep affected by Scrapie and free-ranging Rocky Mountain elk infected with CWD [23–26]. Nevertheless, the precise cellular localization of prions within maternal–foetal interface tissues and their association with specific maternal or foetal cell populations remain poorly characterized. As a result, the cellular mechanisms that may facilitate vertical transmission of cervid prions are unknown.

Reeves’ muntjac, like all cervid species, have a synepitheliochorial, cotyledonary placenta organized into discrete placentomes, where maternal caruncular tissue interfaces with foetal cotyledonary tissue and trophoblasts [27]. The uterine glands examined in this study are located within the maternal endometrium adjacent to the placentome, placing their secretory products in proximity to the foetal trophoblast layer. This organization provides relevant comparative context to the cotyledonary placentomes of sheep, where prion deposition has been reported at the maternal–foetal interface [23, 25].

To further assess mechanisms of CWD vertical transmission, we developed amplification-IHC (AMP-IHC), a novel adaptation of IHC that leverages established tyramide signal amplification (TSA) chemistry to enhance detection sensitivity while preserving tissue architecture and specificity [28, 29]. We optimized AMP-IHC in lymphoid tissue from CWD-infected WTD with low PrP^CWD^ deposition as assessed by conventional IHC. AMP-IHC was then applied to tissues of the pregnancy microenvironment. Using this method, we identified prion accumulation in the uterine glandular epithelium and lumen at the maternal–foetal interface of experimentally infected muntjac, thus providing initial insight into mechanisms of CWD vertical transmission.

## Methods

### Tissues

#### WTD tissues

WTD were sourced through a collaboration with the Warnell School of Forestry and Natural Resources, University of Georgia, and housed in the CWD facility at Colorado State University (CSU). Standardized protocols to prevent unintended exposure and cross-contamination between study cohorts were implemented as previously described [30]. WTD were handled in strict accordance with guidelines for animal care and use provided by the United States Department of Agriculture (USDA), National Institutes of Health (NIH) and the Association for Assessment and Accreditation of Laboratory Animal Care International (AAALAC) with all animal work approved by the CSU Institutional Animal Care and Use Committee (IACUC approval numbers 1466 and 13-4610A).

Lymphoid (mesenteric and retropharyngeal) tissues from CWD-positive (*n*=2) and CWD-negative (*n*=2) WTD were obtained from: (1) A bioarchived aerosol CWD study [31] and (2) A 12 month study of WTD inoculated *per os* (PO) with either 0.5 mg CWD-positive brain homogenate or 0.5 mg of CWD-negative obex and retropharyngeal lymph node homogenate (1.0 mg total).

Experimentally inoculated WTD tissues were used as controls to optimize AMP-IHC due to their known CWD status and extensive characterization. Experimental infection studies in muntjac demonstrate that CWD susceptibility and tissue-level pathogenesis closely mirror those observed in WTD, with prion accumulation occurring in lymphoid tissues early in infection and subsequent involvement of neurotropic tissues, consistent with the tissue distribution reported in WTD [9].

#### Reeves’ muntjac tissues

Reproductive tissues (placentomes and endometrium) from muntjac [CWD-positive (*n*=8); CWD-negative (*n*=2)] were derived from bioarchived CWD vertical transmission studies conducted at CSU [9]. Tissues were collected from animals representing early, mid and late gestation within the ~210-day muntjac pregnancy. Muntjac were experimentally inoculated as described in Table 1. Muntjac for these studies were provided by and transported to CSU by Cervid Solutions Inc, Tellico, TN, USA. Muntjac were bred in our captive cervid facilities located at CSU and were handled in strict accordance with guidelines for animal care and use provided by the USDA, NIH and AAALAC with all animal work approved by the CSU IACUC (approval numbers 02-151A, 08-175A and 11-2615A). Standardized protocols to prevent unintended exposure and cross-contamination between study cohorts were implemented as previously described [30].

**Table 1.** Summary of AMP-IHC findings in muntjac at the maternal–foetal interface. CWD-positive (*n*=8) muntjac were evaluated for prion deposition in the uterine layers surrounding the placentome, with CWD-naïve (*n*=2) animals serving as negative controls. PrP^CWD^ deposition was detected in the surrounding uterine layer of four of eight (50%) CWD-positive muntjac, specifically within the uterine glandular epithelium and luminal spaces. Corresponding amyloid seeding assay data for these tissues have been previously reported [11]

Dam ID	Uterine gland positivity (AMP-IHC)	Uterine gland positivity (IHC)	Clinical disease status	Months post-infection (p.i.)*	Gestational age (no. of months)^†^	Inoculum (route/dose)
MJ 84	2/3^‡^	0/3^‡^	Sub	5	7	CWD+ (0.5 g PO; 0.35 g SQ)
MJ 77	1/2^‡^	0/2^‡^	Sub	12	3	CWD+ (0.5 g PO, 0.5 g SQ)
MJ 73	2/3^‡^	0/3^‡^	Sub	12	3	CWD+ (0.5 g PO, 0.5 g SQ)
MJ 15	1/1^‡^	0/1^‡^	Late	26	NP	CWD+ (2.0 g PO; 0.5 g SQ)
MJ 45	0/4^‡^	0/4^‡^	Early	22	4.8	CWD+ (0.5 mg PO, 0.35 g SQ)
MJ 47	0/5^‡^	0/5^‡^	Early	20	>4.5	CWD+ (0.5 mg PO, 0.35 g SQ)
MJ 19	0/3^‡^	0/3^‡^	Sub	7	3.5	CWD+ (1.5 g PO; 0.35 g SQ)
MJ 11	0/1^‡^	0/1^‡^	Late	25	NP	CWD+ (1.0 g PO)
MJ 20	0/2^‡^	0/2^‡^	n/a	n/a	NP	Not inoculated
MJ 80	0/3^‡^	0/3^‡^	n/a	n/a	>4.5	Not inoculated

N/A denotes CWD-naïve.

NP denotes not pregnant.

Sub denotes subclinical disease status.

Early denotes early signs of CWD (weight loss).

Late denotes euthanized due to terminal clinical CWD disease, with symptoms of hypersalivation, weight loss and ataxia.

* Terminal CWD clinical disease in experimentally inoculated muntjac dams manifests between 23 and 26 months p.i.

† The full-term gestational period for Reeves’ muntjac deer is 7 months.

‡ Indicates the number of independent tissue blocks examined; all glandular profiles present within each available tissue section were evaluated.

### Conventional IHC

Conventional IHCs were run in accordance with a previously published protocol []. In short, tissues were fixed in 10% neutral buffered formalin or paraformaldehyde/lysine/periodate for 5 or 2 days, respectively, and stored in 60% ethanol, embedded in paraffin and sectioned at 5 µm for staining. Slides were de-paraffinized through a series of xylene/ethanol baths, rinsed in running water for 10 min, digested with Proteinase K (PK) (20 mg ml^−1^) at 37 °C in CaCl_2_ Tris Buffer (1 mM CaCl_2_, 50 mM Tris in 1 l of ddH_2_O, pH 7.6) for 30 min, treated with 88 % formic acid for 5 min, and then subjected to a 15 min antigen-retrieval process in 10 mM EDTA (2.9225 g EDTA in 1 l ddH_2_O) at 121 °C. Briefly, slides were quenched for endogenous peroxidase activity with 3% H_2_O_2_ in methanol (30 min), blocked with blocking buffer (Akoya Biosciences; cat#NEL700A001KT) (30 min), incubated with a 1:750 dilution of BAR-224 (Cayman Chemical) in blocking buffer (Akoya) (overnight), bound with the EnVision+Horseradish peroxidase (HRP) anti-mouse system (Dako) secondary antibody (30 min), developed with chromogen AEC (3-amino-9-ethylcarbazole; Abcam; cat#64252) (4 min) and counterstained with haematoxylin (Agilent; cat#S330930-2) and bluing reagent [0.1 g sodium bicarbonate (Merck; cat#3SX0320-1) in 100 ml ddH2O] (4 min each). The use of a 1 : 750 BAR-224 dilution was based on the previously published and optimized conventional IHC protocol for CWD detection [32].

### AMP-IHC

Tissues were prepared as mentioned for conventional IHC with the addition of biotin, conjugated streptavidin-HRP and tyramide substrate (Akoya Biosciences; cat#NEL700A001KT). Tissues were quenched for endogenous peroxidase activity and blocked as in conventional IHC, then incubated overnight with BAR-224 (1 : 8,000; Cayman Chemical) in blocking buffer and subsequently with EnVision+HRP anti-mouse secondary antibody (Dako) for 30 min. Signal was amplified with biotin (1 : 50; 7 min) followed by streptavidin-HRP (1 : 100; 30 min), with washes in 1× TBS (24 g Tris, 88 g NaCl in 1 l ddH_2_O, pH 7.6) between steps. No dedicated endogenous biotin-blocking step was performed. Slides were developed with AEC chromogen (4 min) and counterstained with haematoxylin and bluing reagent in 100 ml (4 min each). The BAR-224 concentration used for AMP-IHC was empirically optimized during assay development by evaluating multiple antibody dilutions to identify a concentration that provided robust PrP^CWD^ immunoreactivity while minimizing nonspecific background in the presence of biotin-TSA. Based on this optimization, a 1 : 8,000 dilution was selected for subsequent AMP-IHC analyses.

### Analysis

Tissue sections were processed side-by-side under identical conditions to allow direct comparison of staining patterns and intensity. Negative-control sections in which the primary antibody was omitted were included to assess nonspecific background staining. Immunoreactivity was assessed qualitatively by light microscopy and categorized relative to background and negative-control tissues as absent, weak, moderate or strong. Only staining that was spatially consistent with tissue architecture and absent in the corresponding negative-control sections was considered specific. Slides from both conventional IHC and AMP-IHC assays were reviewed by a board-certified veterinary anatomic pathologist to confirm staining patterns and diagnostic interpretation. No nonspecific background staining was observed in the primary-antibody omission controls.

## Results

We first evaluated the performance of AMP-IHC relative to conventional IHC for detecting prion deposition in mesenteric and retropharyngeal lymph nodes collected from WTD that were experimentally aerosol- or PO-inoculated with CWD.

**Fig. 1.**
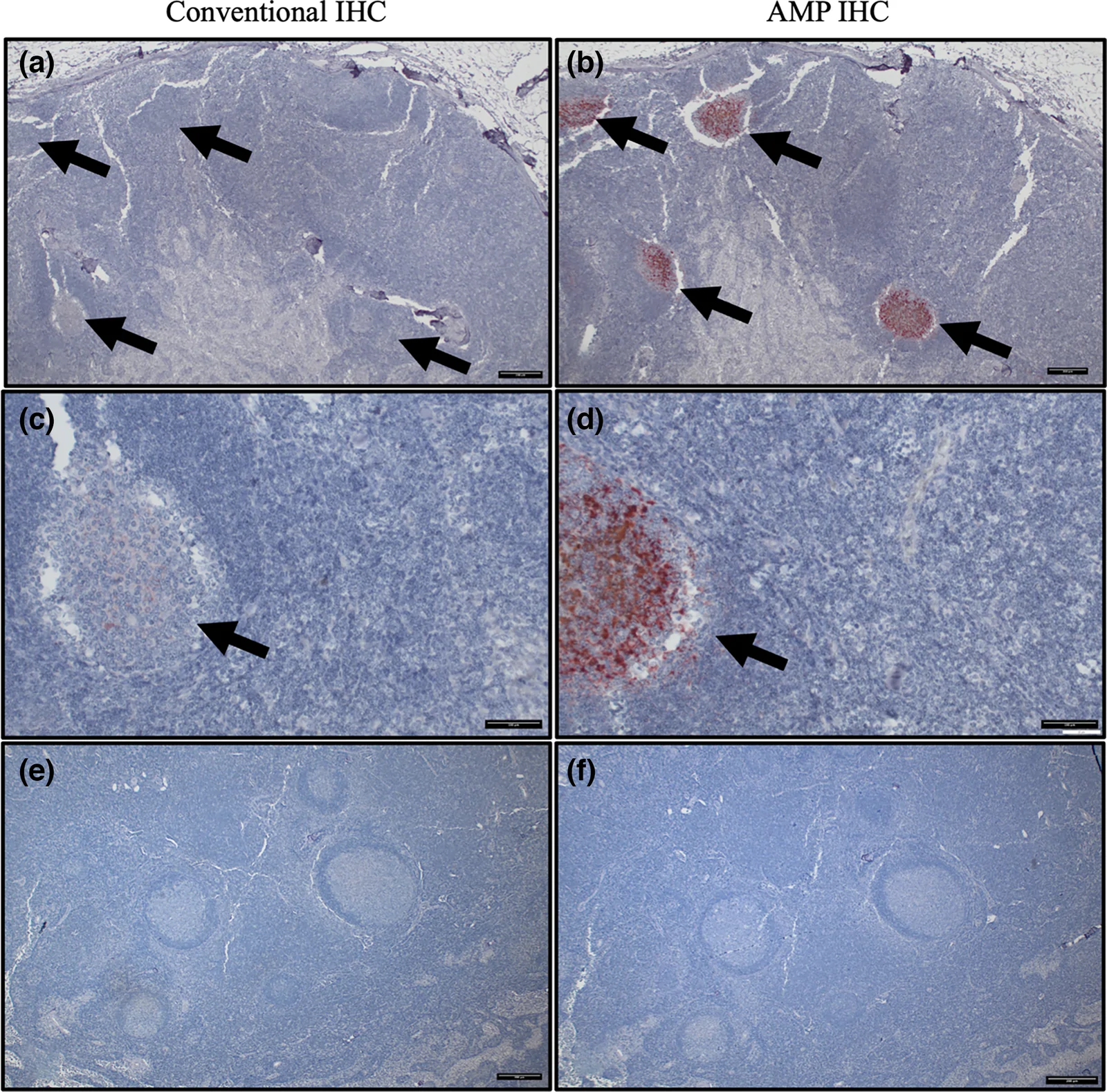
Enhanced detection of PrP^CWD^ by AMP-IHC compared to conventional IHC in mesenteric lymph nodes of WTD. Representative sections of mesenteric lymph node from CWD-positive WTD were stained with BAR-224 antibody using conventional IHC (**a, c**) or AMP-IHC (**b, d**). Sections e and f represent mesenteric lymph node from a CWD-negative WTD stained using conventional IHC (**e**) or AMP-IHC (**f**) with the same antibody. Panels a, b, e and f were imaged at 4× magnification, and panels c and d were imaged at 20× magnification. Arrows indicate PrP^CWD^ deposition. Arrows denote lymphoid follicles with PrP^CWD^ deposition. Scale bars for panels a, b, e, and f=200 µm; scale bars for panels c and d=100 µm.

At low magnification (4×), conventional IHC demonstrated only faint and sparse immunoreactivity within mesenteric lymphoid follicles (Fig. 1a), indicating limited sensitivity in the detection of low-abundance prion deposition under these exposure conditions. In contrast, AMP-IHC applied to the sequential tissue section from the same animal and imaged at identical magnification revealed markedly increased signal intensity, with strong and readily appreciable staining throughout follicular regions (Fig. 1b). Higher magnification (20×) analysis further emphasized these differences. Conventional IHC revealed weak, punctate staining patterns (Fig. 1c). Conversely, AMP-IHC produced robust, well-defined immunoreactivity with substantially enhanced signal intensity and distribution in the same anatomical regions (Fig. 1d). To confirm staining specificity, mesenteric lymph node from a CWD-negative WTD was processed in parallel as a negative control. Conventional IHC performed on this tissue demonstrated no detectable PrP immunoreactivity (Fig. 1e). Similarly, AMP-IHC applied to a sequential section from the same CWD-negative animal also showed no staining (Fig. 1f), indicating the absence of nonspecific signal amplification under these conditions. Results from retropharyngeal lymph node (RPLN) tissues demonstrated the same increase in robust staining using AMP-IHC in comparison to conventional IHC.

Collectively, these findings demonstrate that AMP-IHC markedly enhances the detection of PrP^CWD^ immunoreactivity compared to conventional IHC, allowing clearer visualization of lymphoid PrP^CWD^ deposition following CWD exposure.

Following optimization of AMP-IHC in lymphoid tissues, we next applied this approach to evaluate PrP^CWD^ deposition in uterine tissues from experimentally inoculated muntjac. A total of 10 animals were evaluated, including CWD-infected muntjac (*n*=8) and CWD-naïve muntjac (*n*=2) serving as negative controls. AMP-IHC was used to assess prion accumulation at the maternal–foetal interface. While evaluating placentome samples for PrP^CWD^ deposition, immunoreactivity was identified within the uterine glandular epithelium, prompting expanded evaluation of glandular regions present within these areas of tissue.

**Fig. 2.**
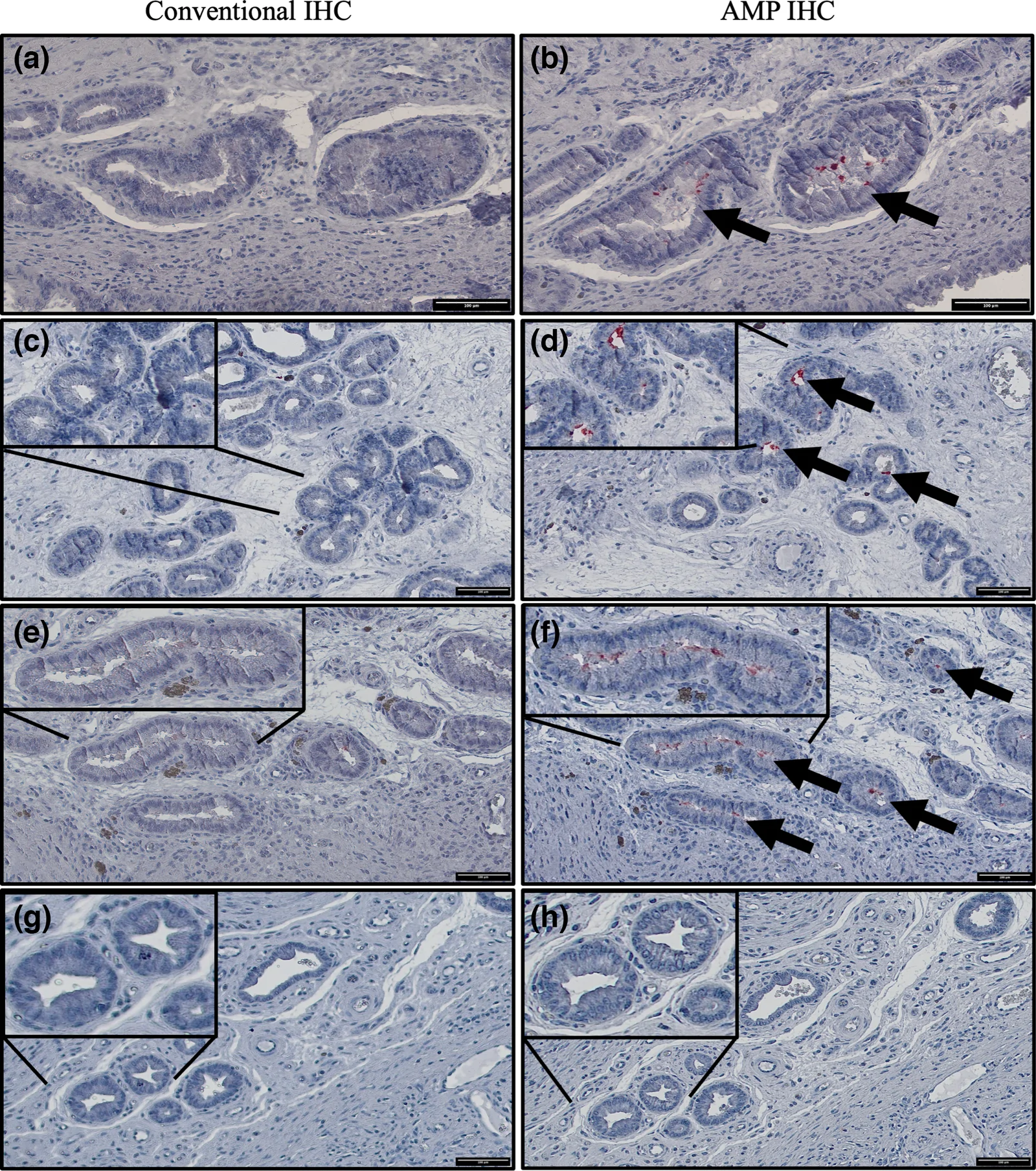
Enhanced detection of PrP^CWD^ by AMP-IHC compared to conventional IHC in uterine glands of Reeves’ muntjac. Representative placentome sections containing uterine glands were stained with BAR-224 antibody using conventional IHC (**a, c, e, g**) or AMP-IHC (**b, d, f, h**). Panels a–f show uterine glands in placentome sections from CWD-infected muntjac, whereas panels g and h show matched tissues from CWD-naïve muntjac serving as negative controls. Panels a and b were imaged at 20× magnification, panels c, d, e, f, g and h at 10× magnification. Arrows indicate PrP^CWD^ deposition. Arrows denote regions of uterine gland with PrP^CWD^ deposition. All scale bars=100 µm.

Using AMP-IHC, prion immunoreactivity (PrP^CWD^ deposition) was visualized in the uterine glands of four of eight (50%) CWD-infected muntjac; notably, zero of eight (0%) of the same CWD-infected muntjac had visible prion immunoreactivity using conventional IHC (Fig. 2, Table 1). All identifiable glandular profiles present within the available tissue sections were evaluated. Staining was distinct and confined to glandular epithelial cells and luminal contents, with surrounding stromal tissues remaining free of PrP^CWD^ deposition. The remaining four CWD-infected animals showed no detectable PrP^CWD^ deposition in comparable regions under identical staining conditions, similar to the two CWD-naïve negative control animals. No apparent relationship between glandular PrP^CWD^ immunoreactivity and gestational age, time post-inoculation or clinical disease status was observed; however, the small cohort precluded meaningful statistical evaluation of these variables.

Direct comparison between conventional IHC and AMP-IHC highlighted the increased sensitivity of the amplified approach. In three representative animals, conventional IHC failed to detect PrP^CWD^ deposition within uterine tissues (Fig. 2a, c, e). Examination of sequential tissue sections from the same animals by AMP-IHC revealed definitive PrP^CWD^ deposition within the uterine glandular epithelium and glandular lumen (Fig. 2b, d, f). PrP^CWD^ deposition revealed by AMP-IHC was robust and well-defined, contrasting sharply with the absence of deposition detection as assessed by conventional IHC methods. Importantly, negative control animals processed in parallel demonstrated no detectable immunoreactivity within uterine glands when assessed by AMP-IHC (Fig. 2g, h). These findings confirm the specificity of AMP-IHC in its ability to detect PrP^CWD^.

Together, these findings demonstrate that AMP-IHC enables sensitive and specific detection of PrP^CWD^ deposition within the uterine glandular structure, revealing previously undetected site- and cellular-specific prion accumulation at the maternal–foetal interface of CWD-infected muntjac.

## Discussion

Here, we provide the first direct histological evidence of PrP^CWD^ deposition (i.e. CWD prions) within the uterine glandular epithelium at the maternal–foetal interface in cervids. This finding is particularly noteworthy given the central role of uterine glands in pregnancy, as they produce histotroph – a complex mixture of nutrients, proteins, lipids, cytokines and growth factors that supports conceptus survival and development prior to and during placentation [33, 34]. Histotroph is released into the uterine lumen, where it is in continuous contact with trophoblast cells, positioning the glandular epithelium as a primary route of molecular exchange between maternal tissues and the developing foetus. The presence of prions within glandular epithelial cells and luminal secretions provides a direct and biologically plausible pathway for foetal exposure. Because histotroph is actively internalized by trophoblasts, it could be hypothesized that prions associated with these secretions may be taken up via endocytosis or extracellular vesicle-mediated mechanisms, enabling trafficking across the placental barrier and access to foetal tissues [35, 36]. Biologically, even low-level prion deposition in the uterine glands may hold significance, as early gestational exposure may lead to prion uptake and amplification within foetal tissues. However, the detection of PrP^CWD^ immunoreactivity by AMP-IHC does not independently establish the presence of infectious prions or demonstrate that the detected material is capable of transmission. These observations highlight the potential for vertical transmission to act as a subtle but persistent driver of CWD maintenance within endemic populations, complementing horizontal and environmental routes of infection. These results build upon our prior studies that demonstrated the presence of prions in reproductive and foetal tissues of infected cervids [9, 10, 14]. They also further support *in utero* CWD exposure during gestation and reveal early targets for maternal–foetal trafficking mechanism(s) investigations.

Previous studies using amyloid seeding assays (RT-QuIC, PMCA) and bioassays confirmed the presence of prions in reproductive tissues of CWD-infected cervids [9–12, 14]. Our findings align with these reports and enhance these findings by providing a cellular mechanism for CWD vertical transmission. AMP-IHC couples molecular sensitivity with histological resolution to reveal the spatial context of prion localization. This methodological advancement highlights the utility of AMP-IHC for investigations involving tissues with presumed low CWD burden, helping to refine our understanding of prion dissemination pathways, especially those associated with expanding our understanding of early prion trafficking within the host.

Our findings are supported by similar reports of prion accumulation at the maternal–foetal interface in other species. Studies in sheep naturally exposed to scrapie have demonstrated immunohistochemical prion deposition within placentomes, indicating a direct association at the maternal–foetal interface [23–25]. Likewise, research in Rocky Mountain elk naturally exposed to CWD has shown prion accumulation in the ovaries, placentomes and uterus, highlighting reproductive tissues as sites of cellular-level prion localization; notably, these studies did not describe the presence of prion association with uterine glands [26]. These findings across species reinforce the biological plausibility of vertical transmission via uterine and placental structures, further supporting our results in muntjac. Combined, they suggest that maternal reproductive tissues can serve as reservoirs and conduits for prion trafficking to the developing foetus.

Collectively, experimental and field-based studies now provide converging evidence that CWD vertical transmission occurs in multiple cervid species. In muntjac, maternal infection results in offspring that develop clinical disease, and bioassay of maternal tissues has demonstrated infectivity [9, 11]. Complementary findings in elk have identified amplifiable prions within tissues of the maternal–foetal interface using PMCA, a signal that correlates strongly with infectivity [10]. More recently, detection of CWD in free-ranging WTD offspring further supports vertical transmission as a biologically relevant component of CWD transmission dynamics under natural exposure conditions [14].

Despite this growing body of evidence, important questions remain regarding the relationship between prion detectability and transmission potential. While immunohistochemical detection of PrP^CWD^ at the maternal–foetal interface provides strong evidence of *in utero* exposure, the demonstration of PrP^CWD^ deposition identified by IHC alone may not reflect infectious potential. Reproductive tissues examined in the present study originated from a previously characterized experimental cohort in which prion seeding activity was evaluated using amplification-based assays [11]. However, the present study was designed to characterize the histological localization of PrP^CWD^ using AMP-IHC rather than to reassess prion seeding activity. The archived material available for the present histological analyses consisted of tissue sections and was therefore not suitable for repeat RT-QuIC or sPMCA analysis of the same tissue material. Consequently, direct concordance between AMP-IHC positivity and amplification-assay results could not be established at the level of the same tissue section or lesion. Thus, AMP-IHC positivity in uterine glands should be interpreted as evidence of localized PrP^CWD^ immunoreactivity rather than definitive evidence of infectious prions. Future studies combining AMP-IHC with RT-QuIC, PMCA or bioassay using matched tissue samples will be important for determining whether the cellular deposits identified by AMP-IHC correspond to biologically active prion material. Continued investigation of free-ranging cervid populations across diverse geographic regions that include bioassay of reproductive tissues will be essential to fully define the contribution of vertical transmission to CWD ecology and disease spread.

An additional limitation of this study is the small number of archived animals available for histological evaluation. Of the eight CWD-positive animals examined, four exhibited PrP^CWD^ immunoreactivity within uterine glandular epithelium, whereas four did not. The biological basis for this binary pattern of detection remains unclear. Potential sources of inter-animal variation include disease stage, time post-inoculation, gestational stage, individual variation in prion tissue distribution and overall prion burden. However, the small cohort size and limited representation of these variables preclude meaningful statistical evaluation or identification of predictors of uterine gland PrP^CWD^ deposition. Therefore, these findings should be interpreted descriptively, and larger, systematically sampled cohorts will be necessary to determine the prevalence of uterine gland PrP^CWD^ deposition and establish the biological factors associated with its occurrence.

Longitudinal monitoring of offspring born to CWD-positive dams under controlled, captive experimental conditions has demonstrated that maternal exposure can result in persistent infection, as shown in muntjac [9]. More recently, evidence of vertical transmission under natural exposure conditions has been reported in free-ranging WTD, establishing ecological relevance [14]. Building upon these findings, future studies should extend longitudinal surveillance to additional free-ranging cervid populations across diverse geographic regions, including western North America, Canada and Scandinavia, to assess species- and environment-specific influences on vertical transmission dynamics.

AMP-IHC may be of additional use for evaluation of low-burden tissues to enhance early detection of prions and inform surveillance strategies. Moreover, applying AMP-IHC in parallel with ultrasensitive prion amplification assays, such as RT-QuIC and PMCA, may strengthen validation of prion presence and facilitate improved characterization of prion trafficking and transmission mechanisms. Importantly, incorporation of AMP-IHC into existing USDA-approved diagnostic laboratories, where conventional IHC is the current gold standard for prion disease surveillance, could represent a practical and scalable enhancement. Because AMP-IHC builds upon established IHC workflows, adoption would likely require minimal modification to existing protocols while providing improved signal detection, thereby increasing diagnostic sensitivity and robustness without fundamentally altering current testing infrastructure.

An important methodological consideration is the difference in BAR-224 antibody dilution between conventional IHC and AMP-IHC. Conventional IHC was performed using the previously optimized 1 : 750 dilution, whereas AMP-IHC was optimized using a substantially lower antibody concentration (1 : 8,000). Notably, AMP-IHC produced stronger immunoreactivity despite the ~10-fold lower primary antibody concentration used for AMP-IHC. This observation supports the contribution of the biotin, conjugated streptavidin-HRP and tyramide substrate-based amplification system to the increased signal intensity observed with AMP-IHC, rather than an effect attributable simply to increased primary antibody concentration. Higher primary antibody concentrations were also evaluated during AMP-IHC optimization but resulted in increased nonspecific background staining, limiting their utility despite the potential for increased specific signal. Because the assays were not directly compared using identical antibody concentrations, the independent contribution of antibody concentration cannot be completely excluded. Thus, the present study compares the performance of the respective optimized conventional IHC and AMP-IHC protocols. In addition, here, we evaluated immunoreactivity qualitatively rather than using quantitative or semi-quantitative measures of signal intensity, deposit density or the proportion of affected glandular profiles. Although the qualitative assessment demonstrated stronger immunolabeling with AMP-IHC, quantitative image analysis or standardized scoring would provide a more objective measure of the magnitude of signal enhancement and tissue involvement and should be considered in future studies.

Our findings underscore the importance of reproductive tissues in prion ecology. Localization of prions within the uterine glandular epithelium identifies a potential cellular reservoir facilitating maternal–foetal transfer and provides a plausible mechanism for CWD persistence in endemic areas where environmental transmission alone cannot explain disease maintenance [37]. Consideration of reproductive and perinatal pathways in disease modelling, surveillance and management may therefore be warranted.

Together, these findings establish a mechanistic framework for vertical transmission in which prions accumulate within uterine glands, are secreted into the uterine lumen as components of histotroph and are subsequently encountered and internalized at the maternal–foetal interface. This sequence provides a direct maternal-to-foetal transmission route that does not rely solely on haematogenous spread. By identifying uterine glands as both a site of prion deposition and a functional conduit for transfer, this work refines the biological basis of vertical transmission and highlights a previously underappreciated pathway that may contribute to prion persistence in cervid populations.

## Abbreviations

AAALAC: Association for Assessment and Accreditation of Laboratory Animal Care International
AEC: 3-amino-9-ethylcarbazole
AMP-IHC: amplification-IHC
CSU: Colorado State University
CWD: chronic wasting disease
HRP: Horseradish peroxidase
IACUC: Institutional Animal Care and Use Committee
IHC: immunohistochemistry
NIH: National Institutes of Health
p.i.: post-infection
PMCA: protein misfolding cyclic amplification
PO: *per os*
PrP: prion protein
RT-QuIC: real-time quaking-induced conversion
sPMCA: serial protein misfolding cyclic amplification
TSA: tyramide signal amplification
USDA: United States Department of Agriculture
WTD: white-tailed deer
